# A Comparison of the Validities of Traditional Chinese Versions of the Work Productivity and Activity Impairment Questionnaire: General Health and the World Health Organization’s Health and Work Performance Questionnaire

**DOI:** 10.3390/ijerph19074417

**Published:** 2022-04-06

**Authors:** Kim-Ngan Ta-Thi, Kai-Jen Chuang

**Affiliations:** 1 School of Public Health, College of Public Health, Taipei Medical University, Taipei 110, Taiwan; d508108005@tmu.edu.tw; 2 Faculty of Public Health, University of Medicine and Pharmacy at Ho Chi Minh City, Ho Chi Minh City 700000, Vietnam; 3 Department of Public Health, School of Medicine, College of Medicine, Taipei Medical University, Taipei 110, Taiwan

**Keywords:** validity, productivity, WPAI:GH, traditional Chinese, WHO-HPQ, occupational health

## Abstract

There is a lack of valid instruments for measuring productivity loss due to illness. This study aimed to compare the validities of traditional Chinese versions of the Work Productivity and Activity Impairment: General Health (C-WPAI:GH) and the World Health Organization’s Health and Work Performance Questionnaire (C-WHO-HPQ), and to define the factors associated with productivity loss. We conducted a cross-sectional study of 165 Taiwanese employees in technology companies. Spearman’s correlation coefficients and ANOVAs were used to test the validities of the C-WPAI:GH and C-WHO-HPQ. Bayesian model averaging was used for multiple linear regression to define the factors related to productivity loss. The C-WPAI:GH had acceptable validities for assessing the productivity loss of Taiwanese employees. The C-WHO-HPQ had acceptable content validity and concurrent criterion validity. However, the construct validity of the C-WHO-HPQ was insufficient (less than 75% of results were consistent with our hypotheses). Absenteeism in the C-WPAI:GH was associated with education, physical functioning and job satisfaction. There were significant associations of bodily pain, social functioning and general health with presenteeism, overall work impairment and activity impairment in the C-WPAI:GH. A linear correlation was found between education and activity impairment in the C-WPAI:GH. The C-WPAI:GH can be used to evaluate productivity loss due to illness.

## 1. Introduction

Work productivity is one of the most important resources contributing to organizational development and also sustainable national development. Assessing work performance is an issue of concern for employers. By assessing this information, employers have data to develop policies on promotions, incentive bonuses and salary increases.

Health problems act as a deterrent to employee productivity. To measure productivity loss due to illness, many researchers use two concepts: absenteeism and presenteeism. Absenteeism means that employees are away from their work due to sickness or disability. Presenteeism means productivity loss due to the sickness or medical conditions of employees who are present at work but not performing their duties at full competence [1,2,3].

Many studies have been conducted to estimate absenteeism and presenteeism. The absenteeism cost due to smoking by Taiwanese employees is estimated to be USD 184 million every year [4]. In a self-reported productivity questionnaire, the average presenteeism score of Vietnamese patients in a methadone maintenance program was estimated to be 31.5% and was converted into a mean weekly presenteeism cost of USD 8.7 [5]. In a cross-sectional study in Iranian hospitals, the average absenteeism days of 1958 healthcare workers who had COVID-19 in 2021 was 16.44 days and it was equivalent to USD 671.4 per person [6]. However, using a health measure with limited evidence on its validity when estimating absenteeism and presenteeism might increase the bias of the results.

To date, many measurement tools of work productivity have been developed. A key issue is that a valid instrument on productivity loss due to health problems might help managers and policymakers better evaluate the economic burden of illness and the cost-effectiveness of health interventions. In a systematic review of 21 instruments measuring presenteeism by the Institute of Health Economics (Edmonton, AB, Canada) in 2015 [7], the Work Productivity and Activity Impairment Questionnaire: General Health (WPAI:GH) and the World Health Organization’s Health and Work Performance Questionnaire (WHO-HPQ) (short form) were two of the most popular instruments because they are short and available online. In addition, these scales can be used for the general population, general health and monetization of lost productivity. Although these two scales have some limitations [8], the WPAI:GH and WHO-HPQ were validated and have been used commonly in papers published during 2018–2021 according to the newest systematic review [9].

There is limited evidence on the validity and reliability of these instruments [10]. In 1993, Reilly et al. [11] developed the quantitative work productivity and activity impairment (WPAI) questionnaire. The WPAI was correlated with the Short Form-36 (SF-36) and the self-reported severity of health problems, which proved the construct validity of the WPAI. The Portuguese version of the WPAI:GH was associated with the SF-36 in a validation study on 100 Brazilian employees and patients [12]. Other validation studies of the WPAI:GH were carried out in English-speaking countries, such as the UK (2010) and Singapore (2020) [13,14]. It was found that, until now, the WHO-HPQ (short form) has been both translated and validated only in Japan in 2020 [15]. Overall, according to a systematic review in 2015 [10], the WPAI:GH and WHO-HPQ were proved to have acceptable construct validity and concurrent validity. To our best knowledge, data on employees’ work performance and related factors are still deficient because valid instruments for this use in Taiwan are lacking. In addition, although health problems affect employee productivity, there is limited evidence on the models or mapping algorithms for predicting productivity loss using measures of health status [16]. Therefore, this study aimed (i) to compare the validities of the traditional Chinese versions of the WPAI:GH and WHO-HPQ in Taiwanese employees in technology companies in northern Taiwan and (ii) determine the associations of demographics, health status, job satisfaction, and disability status with productivity loss.

## 2. Materials and Methods

### 2.1. Process of Translation

We contacted the authors of the WHO-HPQ and WPAI:GH. There were no terms and conditions on translating these questionnaires for use in our validation study.

We followed Sousa’s guidelines for the process of translation [17]: forward translation, back-translation and an expert panel. Two independent translators translated the WHO-HPQ from English into traditional Chinese. For the WPAI:GH, we obtained one traditional Chinese version from the website of the WPAI:GH’s author [18] and another version translated from English to traditional Chinese by a translator. Another independent translator synthesized the two versions of these scales into the preliminary initial traditional Chinese versions.

Two other independent translators translated the traditional Chinese version back into English and another third independent translator synthesized the English back-translated version. Next, two independent native English-speaking researchers compared the back-translated version with the original English version to define any differences in meaning.

After that, we conducted two sessions with panels of experts in the field of health economics and occupational health. We emailed four experts in the first session to obtain independent ratings of the content validity from each expert, especially regarding the clarity, relevance and translation equivalence of the traditional Chinese versions. In the second face-to-face expert panel meeting, three experts discussed and reached a consensus on some minor revisions to finalize the translated versions (Supplementary Material).

### 2.2. Instruments

Table 1 describes information of the Work Productivity and Activity Impairment Questionnaire: General Health (WPAI:GH) and the World Health Organization’s Health and Work Performance Questionnaire (WHO-HPQ).

The health status was measured by the 36-Item Short Form Survey (SF-36) Version 1.0 (RAND 36-Item Health Survey 1.0 Questionnaire). The SF-36 includes 36 questions on eight domains: physical functioning, bodily pain, general health, vitality, social functioning, role limitations due to physical problems (role—physical), role limitations due to emotional problems (role—emotional) and mental health. Higher scores represent better health outcomes [20].

Job satisfaction was assessed by one item: “Overall, how satisfied are you with your job?” on a five-point Likert scale from 1 to 5, with five respective categories as follows: “completely dissatisfied”, “moderately dissatisfied”, “neither satisfied nor dissatisfied”, “moderately satisfied” and “completely satisfied” [21].

Disability status was classified into work-related disability, non-work-related disability and no disability [21].

### 2.3. Sample

A cross-sectional study was conducted in 10 technology companies in northern Taiwan. Among 3308 employees, we recruited 165 study participants by random sampling with the following inclusion criteria: (i) a Taiwanese employee, (ii) at least 20 years of age, (iii) the ability to read and speak Chinese, (iv) having a full-time job, (v) no missing data on the WHO-HPQ and WPAI:GH questionnaires, and (vi) agreeing to join this study. Informed consent was obtained from each participant before the employees were interviewed.

### 2.4. Validities

Based on the results of the COSMIN study about consensus on the definitions of psychometric properties for health measurement instruments [22], we present the content validity, construct validity and criterion validity.

(1) Content validity: we emailed four experts two questions to evaluate the content validity of the traditional Chinese versions of the WPAI:GH (C-WPAI:GH) and the WHO-HPQ (C-WHO-HPQ): “Has the translated version kept the same meaning as the original English version?” and “Is the translated version clear and easy to understand for the general Taiwanese population?”. Possible answers were: (1) strongly disagree, (2) disagree, (3) neutral, (4) agree, and (5) strongly agree.

(2) Construct validity: We examined the construct validity by testing hypotheses on the correlates of the C-WHO-HPQ and C-WPAI:GH with another instrument (the SF-36), and discrepancies between relevant groups (job satisfaction and disability status).

(3) Criterion validity: Because there is no gold standard to measure productivity loss due to illness, the C-WHO-HPQ scores were compared with the C-WPAI:GH scores for concurrent criterion validity between two similar instruments.

### 2.5. Data Analysis

Data were analyzed by R Version 4.1.2. Shapiro–Wilk’s test and histograms were used to define whether the continuous variables were normally distributed. We assumed that missing values were missing at random and used the multiple imputation method to handle missing data.

To evaluate the construct validity, we formed hypothesies based on previous validation studies [11,12,13,21], as follows:

- 80 hypotheses for the C-WHO-HPQ: Eight outcomes of the C-WHO-HPQ were correlated with the eight domains of the SF-36, job satisfaction and disability status.
- 40 hypotheses for the C-WPAI:GH: Four outcomes of C-WPAI:GH were correlated with the eight domains of the SF-36, job satisfaction and disability status.

The construct validity was considered adequate if at least 75% of the results were consistent with the hypotheses [23].

We used the Spearman correlation coefficients among the C-WHO-HPQ, C-WPAI:GH, and SF-36, because these variables were not normally distributed. Spearman’s rho values of <0.3, 0.3~0.5 and >0.5 were, respectively, considered weak, moderate and strong relationships [24]. To decrease the risk of a Type I error, we used Bonferroni correction with *p* < 0.0008 (C-WHO-HPQ and SF-36) and *p* < 0.0016 (SF-36 and C-WPAI:GH, and C-WHO-HPQ and C-WPAI:GH) as statistical significance for multiple comparisons [25].

An analysis of variance (ANOVA) was used to test differences in the productivity scores of employees reporting that they had had a work-related disability, a non-work-related disability and no disability in the past month, and the associations of the C-WHO-HPQ and C-WPAI:GH with job satisfaction. The Tukey–Kramer test was calculated for multiple comparisons to determine where the significant differences existed.

We used the Bayesian model averaging approach for the multiple linear regression models to determine the associations among demographic information, health status, job satisfaction and productivity loss [26,27]. It was shown that this approach performed better than stepwise regression of the frequentist approach [28,29]. The regression coefficient (β) and 95% credible interval (95% CrI) [30] were computed under the non-informative reference prior by using the BAS package in R software [31,32].

## 3. Results

### 3.1. Descriptive Statistics

Study subjects were aged 22~61 years (mean = 35). The majority of the participants were male (78%). Over half of employees had received a bachelor’s degree or higher (70%). Of the 165 employees who answered the questionnaire, the percentages of each job category were as follows: 15% were managers or professionals (e.g., computer engineers, researchers), 13% were technologists (e.g., nutritionists, electricians), 35% were in an office job or service, 26% were productive technologists who operated machines, and 11% were productive technologists who used their bodies (e.g., cleaning, packaging).

### 3.2. Content Validity

All experts agreed that the C-WPAI:GH kept the same meaning as the original version, and was clear and easily understood by the general Taiwanese population (one out of the four experts strongly agreed and three-fourths of the experts agreed). The response rate was 25% strongly agree, 50% agree and 25% neutral regarding the clarity, relevance and translation equivalence of the C-WHO-HPQ.

### 3.3. Construct Validity

As presented in Table 2, significant correlations were presented, with correlation coefficients and *p* values in bold.

There were significant associations between health status and the C-WPAI:GH scores, especially presenteeism, overall work impairment and activity impairment with physical functioning, role—physical, bodily pain, general health, vitality and social functioning (strong relationships, *r*_s_ values ranged from −0.76 to −0.53, *p* < 0.0001). Only activity impairment had a moderate relationship with role—emotional (*r*_s_ values = −0.33, *p* < 0.0001). Absenteeism was negatively correlated with health status, except for role—emotional and mental health (*r*_s_ values ranged from −0.45 to −0.35, *p* < 0.0001), while presenteeism, overall work impairment and activity impairment were moderately correlated with mental health (*r*_s_ values ranged from −0.44 to −0.36, *p* < 0.0001).

Table 3 presents significant differences between three C-WPAI:GH outcomes (presenteeism, overall work impairment and activity impairment) of three groups of employees who reported that they had had a work-related disability, non-work-related disability or no disability in the past month (*p* < 0.001). Job satisfaction was positively correlated with relative absenteeism (4-week estimate), relative hours of work (4-week estimate) (*p* = 0.004), absolute presenteeism, relative presenteeism (*p* < 0.0001), relative absenteeism (7-day estimates) and relative hours of work (7-day estimates) (*p* = 0.045) in the C-WHO-HPQ. All C-WPAI:GH scores were also correlated with job satisfaction (*p* < 0.0001). However, associations were not found between disability status and any C-WHO-HPQ scores.

In Table S1 (Supplementary Materials), the results of the Turkey–Kramer tests show that subjects who had a work-related disability had significantly higher presenteeism scores (Δ = 23.924, *p* = 0.0001) and overall work impairment scores (Δ = 24.090, *p* = 0.0001) than subjects who had no disability. The activity impairment scores were significantly different between subjects with a work-related disability and those with no disability (Δ = −21.667, *p* = 0.0004). Tables S2 and S3 show the statistically significant differences among the compared groups in detail.

Table 2 and Table 3 show there were 14 (18%) and 35 (88%) correlations, respectively, of the C-WHO-HPQ (80 hypotheses) and C-WPAI:GH (40 hypotheses) with the eight subscales of the SF-36, job satisfaction and disability status. These results indicate the construct validity of the WPAI:GH was sufficient (over 75% of results were consistent with our hypotheses) while the construct validity of the WHO-HPQ was insufficient.

### 3.4. Criterion Validity

Table 4 shows that all C-WPAI:GH outcomes and absolute presenteeism in the C-WHO-HPQ were negatively correlated (*r*_s_ values ranged from −0.48 to −0.32, *p* < 0.0001). All C-WHO-HPQ outcomes and absenteeism measured by the C-WPAI:GH were significantly correlated (*r*_s_ values ranged from −0.47 to 0.47, *p* < 0.0003).

### 3.5. Associations of Demographics, Health Status, Job Satisfaction and Disability Status with Productivity Loss

In Table 5, given these data, there was a 95% chance that presenteeism, overall work impairment and activity impairment scores increased by 0.09 to 0.95, 0.10 to 0.96 and 0.02 to 0.88, respectively, with one additional increase in age. There was a 95% probability that absenteeism and activity impairment scores would increase by 0.22 up to 17.40 and 6.46 up to 53.41 or higher, respectively, if the employees had graduated from senior high school. There was a 95% probability that absenteeism and activity impairment scores would be within 0.47 to 17.79 and 5.62 to 52.95, respectively, if the employees graduated from university, given the data. The 95% credible intervals were (−14.04; −4.09), (−15.90; −5.25), (−15.65; −4.90) and (−16.06; −2.76) when employees were moderately dissatisfied, neither satisfied nor dissatisfied, moderately satisfied and completely satisfied with their jobs, respectively. Correlations were found among bodily pain, social functioning, and general health with presenteeism, overall work impairment and activity impairment.

## 4. Discussion

Employers might pay little attention to productivity loss due to health problems because of a lack of valid and reliable instruments. In our study, the C-WPAI:GH was a valid instrument for assessing the productivity loss of Taiwanese employees. The C-WHO-HPQ had acceptable content validity and concurrent criterion validity. However, the construct validity of the C-WHO-HPQ was insufficient (less than 75% of results were consistent with our hypotheses). There were linear relationships of education, job satisfaction and physical functioning with absenteeism. Linear relationships were found for age, pain, social functioning and general health with presenteeism, overall work impairment and activity impairment. A linear correlation was found between education and activity impairment.

Our study confirms that the C-WPAI:GH is a valid instrument for assessing productivity loss due to illness. However, previous validation studies yielded inconsistent results on the associations between health status and the WPAI:GH [11,14]. Our results of the associations between health status and productivity were inconsistent with those of previous validation studies because the participants in previous studies were patients and older than our study subjects. Another possible explanation for this inconsistency might be that different versions of the SF-36 instrument or other instruments were used to evaluate health status in previous studies. Three measures of the SF-36 instrument (role—physical, role—emotional and pain) were used in the first validation study of the WPAI [11]. The findings of other validation studies on patients with axial spondylarthritis in Singapore and patients with rheumatoid arthritis in the UK differed from those of our study because other instruments were used to measure the health status or specific diseases of those patients [13,14]. A validation study in Brazilian patients showed that absenteeism was not related to the SF-36 instrument, while other WPAI:GH outcomes were correlated with most subscales of the SF-36; particularly good correlations existed with physical functioning, pain and role function (physical) [12]. There were 82% female participants in that study, which might have caused the different results for health status and productivity compared with the 78% male participants in our study.

Contrary to expectations, our study was unable to demonstrate that the construct validity of the C-WHO-HPQ was adequate for our hypotheses to evaluate productivity loss due to health problems. This result may be explained by the fact that the short form of the WHO-HPQ was used in our study, while the long form or the work functioning subscale of the WHO-HPQ was used in previous validation studies [1,33]. Another possible explanation for this could be differences in the age, gender and education of participants and the study designs between our study and another validation study (an online survey in Japan) [15].

There was no significant relationship between any absenteeism scores of the C-WHO-HPQ and presenteeism in the C-WPAI:GH. This finding is consistent with the absenteeism scores of the WHO-HPQ not being associated with the presenteeism scores of other presenteeism scales [15]. In addition, our study confirmed that the presenteeism scores of the C-WHO-HPQ were associated with the scores of other presenteeism scales. A note of caution is due here, since the presenteeism of the WHO-HPQ reflects productivity loss at work for any reason (including health problems) [15].

Our study had similar results with other studies on the associations between physical functioning and absenteeism [34], and also between pain and presenteeism [13]. Physical functioning might cause bad consequences for musculoskeletal conditions, which would result in absenteeism [34]. Pain makes it difficult for employees to concentrate on their work, which means reduced performance at work (presenteeism) [35].

There are several limitations of this study. First, the study participants were recruited in technology companies, so we cannot generalize the results to employees in other fields or businesses. Second, the WPAI:GH and WHO-HPQ are questionnaires based on the number of working hours, which might be prone to recall bias. Third, our study had a cross-sectional design which could not provide cause-and-effect relationships between health and productivity.

Our study suggests that the C-WPAI:GH has acceptable validity for Taiwanese employees in technology companies. A valid instrument for productivity loss due to health problems might help managers and policymakers better evaluate the economic burden of illness and the cost-effectiveness of health interventions. The results of our study also highlight which domains of health status, especially physical functioning, bodily pain, social functioning and general health, were associated with employee productivity.

## 5. Conclusions

In conclusion, the traditional Chinese version of the WPAI:GH is a valid instrument for assessing productivity loss due to illness in Taiwanese employees in technology companies. The C-WHO-HPQ had acceptable content validity and concurrent criterion validity. However, the construct validity of the C-WHO-HPQ was not adequate (less than 75% of the results were consistent with our hypotheses). Employers and policymakers should consider the domains of health status in designing suitable health promotion programs in organizations and treatments in hospitals to reduce production loss because work productivity is an important factor in the sustainability of any organization’s overall performance. A further validation study is suggested to generalize the results on the validity of these two scales for other jobs and organizations.

## Figures and Tables

**Table 1:** Items in the Work Productivity and Activity Impairment Questionnaire: General Health (WPAI:GH) and the World Health Organization’s Health and Work Performance Questionnaire (WHO-HPQ).

	WPAI:GH [11,18]	WHO-HPQ (Short Form) [1,2,19]
Construct	Six questions: Q1: Current employment status; Q2: Hours missed because of illness; Q3: Hours missed for other reasons; Q4: Hours actually worked; Q5: Degree of illness that affected productivity while working; Q6: Degree of illness that affected regular activities.	Five main questions: B3: The number of hours the employee worked in the past 7 days; B4: The number of hours the employer expects the employee to work in a typical 7-day week; B6: The number of hours the employee worked in the past 4 weeks (28 days); B9: How the employee rates the usual performance of most workers in a job similar to his/hers; B11: How the employee rates his/her overall productivity on days he/she worked during the past 4 weeks (28 days).
Scoring	The scores were multiplied by 100 to convert them into percentages. The WPAI:GH has four outcomes as follows: (1) Absenteeism = Q2/(Q2 + Q4) × 100; (2) Presenteeism = Q5/10 × 100; (3) Overall work impairment = Q2/(Q2 + Q4) + [(1 − (Q2/(Q2 + Q4))) × (Q5/10)] × 100; (4) Activity impairment = Q6/10 × 100.	The WHO-HPQ has eight outcomes as follows: Absenteeism (Using 4-week estimates): (1) Absolute absenteeism = 4 × B4 − B6; (2) Relative absenteeism = (4 × B4 − B6)/(4 × B4); (3) Relative hours of work = B6/(4 × B4); Absenteeism (Using 7-day estimates): (4) Absolute absenteeism = (4 × B4) − (4 × B3); (5) Relative absenteeism = ((4 × B4) − (4 × B3))/(4 × B4); (6) Relative hours of work = B3/B4; Presenteeism: (7) Absolute presenteeism = 10 × B11; (8) Relative presenteeism = B11/B9
Interpretation	WPAI:GH outcomes estimate impairment percentages, with higher impairment indicating lower productivity.	Higher scores of absenteeism indicate higher amounts of productivity loss (hours lost per month). A higher score of presenteeism indicates a lower amount of productivity loss.

**Table 2:** Associations of the C-WPAI:GH and C-WHO-HPQ with the Short Form (SF-36) using Spearman’s correlation coefficients (*r*_s_ and *p* values).

	C-WPAI:GH							
	Absenteeism		Presenteeism		Overall Work Impairment		Activity Impairment	
Physical functioning	*r*_s_ = −0.39; ***p* < 0.0001**		*r*_s_ = −0.74; ***p* < 0.0001**		*r*_s_ = −0.74; ***p* < 0.0001**		*r*_s_ = −0.68; ***p* < 0.0001**	
Role—physical	*r*_s_ = −0.37; ***p* < 0.0001**		*r*_s_ = −0.73; ***p* < 0.0001**		*r*_s_ = −0.73; ***p* < 0.0001**		*r*_s_ = −0.66; ***p* < 0.0001**	
Bodily pain	*r*_s_ = −0.45; ***p* < 0.0001**		*r*_s_ = −0.73; ***p* < 0.0001**		*r*_s_ = −0.74; ***p* < 0.0001**		*r*_s_ = −0.66; ***p* < 0.0001**	
General health	*r*_s_ = −0.40; ***p* < 0.0001**		*r*_s_ = −0.57; ***p* < 0.0001**		*r*_s_ = −0.57; ***p* < 0.0001**		*r*_s_ = −0.57; ***p* < 0.0001**	
Vitality	*r*_s_ = −0.35; ***p* < 0.0001**		*r*_s_ = −0.54; ***p* < 0.0001**		*r*_s_ = −0.53; ***p* < 0.0001**		*r*_s_ = −0.59; ***p* < 0.0001**	
Social functioning	*r*_s_ = −0.37; ***p* < 0.0001**		*r*_s_ = −0.70; ***p* < 0.0001**		*r*_s_ = −0.70; ***p* < 0.0001**		*r*_s_ = −0.76; ***p* < 0.0001**	
Role—emotional	*r*_s_ = 0.02; *p* = 0.78		*r*_s_ = −0.22; *p* = 0.005		*r*_s_ = −0.21; *p* = 0.006		*r*_s_ = −0.33; ***p* < 0.0001**	
Mental health	*r*_s_ = −0.25; *p* = 0.001		*r*_s_ = −0.36; ***p* < 0.0001**		*r*_s_ = −0.36; ***p* < 0.0001**		*r*_s_ = −0.44; ***p* < 0.0001**	
	**C-WHO-HPQ**							
	**Absolute Absenteeism ^a^**	**Relative Absenteeism ^a^**	**Relative Hours of Work ^a^**	**Absolute Absenteeism ^b^**	**Relative Absenteeism ^b^**	**Relative Hours of Work ^b^**	**Absolute Presenteeism**	**Relative Presenteeism**
Physical functioning	*r*_s_ = −0.03; *p* = 0.70	*r*_s_ = −0.03; *p* = 0.68	*r*_s_ = 0.03; *p* = 0.68	*r*_s_ = −0.01; *p* = 0.88	*r*_s_ = −0.01; *p* = 0.88	*r*_s_ = 0.01; *p* = 0.88	*r*_s_ = 0.33; ***p* < 0.0001**	*r*_s_ = 0.08; *p* = 0.34
Role–physical	*r*_s_ = −0.03; *p* = 0.66	*r*_s_ = −0.04; *p* = 0.64	*r*_s_ = 0.03; *p* = 0.68	*r*_s_ = −0.04; *p* = 0.64	*r*_s_ = −0.04; *p* = 0.64	*r*_s_ = 0.04; *p* = 0.64	*r*_s_ = 0.32; ***p* < 0.0001**	*r*_s_ = 0.12; p = 0.13
Bodily pain	*r*_s_ = −0.07; *p* = 0.38	*r*_s_ = −0.07; *p* = 0.37	*r*_s_ = 0.07; *p* = 0.37	*r*_s_ = −0.05; *p* = 0.53	*r*_s_ = −0.05; *p* = 0.53	*r*_s_ = 0.05; *p* = 0.53	*r*_s_ = 0.29; ***p* = 0.0001**	*r*_s_ = 0.04; *p* = 0.65
General health	*r*_s_ = −0.09; *p* = 0.26	*r*_s_ = −0.09; *p* = 0.25	*r*_s_ = 0.09; *p* = 0.25	*r*_s_ = −0.06; *p* = 0.46	*r*_s_ = −0.06; *p* = 0.45	*r*_s_ = 0.06; *p* = 0.45	*r*_s_ = 0.42; ***p* < 0.0001**	*r*_s_ = 0.27; ***p* = 0.0005**
Vitality	*r*_s_ = −0.06; *p* = 0.43	*r*_s_ = −0.06; *p* = 0.42	*r*_s_ = 0.06; *p* = 0.42	*r*_s_ = −0.06; *p* = 0.44	*r*_s_ = −0.06; *p* = 0.43	*r*_s_ = 0.06; *p* = 0.43	*r*_s_ = 0.45; ***p* < 0.0001**	*r*_s_ = 0.17; *p* = 0.03
Social functioning	*r*_s_ = −0.01; *p* = 0.93	*r*_s_ = −0.01; *p* = 0.90	*r*_s_ = 0.01; *p* = 0.90	*r*_s_ = 0.03; *p* = 0.69	*r*_s_ = 0.03; *p* = 0.70	*r*_s_ = −0.03; *p* = 0.70	*r*_s_ = 0.42; ***p* < 0.0001**	*r*_s_ = 0.18; *p* = 0.02
Role–emotional	*r*_s_ = 0.21; *p* = 0.006	*r*_s_ = 0.21; *p* = 0.006	*r*_s_ = −0.21; *p* = 0.006	*r*_s_ = 0.20; *p* = 0.01	*r*_s_ = 0.20; *p* = 0.009	*r*_s_ = −0.20; *p* = 0.009	*r*_s_ = 0.22; *p* = 0.005	*r*_s_ = −0.02; *p* = 0.81
Mental health	*r*_s_ = −0.04; *p* = 0.59	*r*_s_ = −0.04; *p* = 0.57	*r*_s_ = 0.04; *p* = 0.57	*r*_s_ = −0.03; *p* = 0.69	*r*_s_ = −0.03; *p* = 0.68	*r*_s_ = 0.03; *p* = 0.68	*r*_s_ = 0.35; ***p* < 0.0001**	*r*_s_ = 0.18; *p* = 0.02

^a^ 4-week estimates; ^b^ 7-day estimates.

**Table 3:** Associations of the C-WPAI:GH and C-WHO-HPQ with disability status and job satisfaction (mean ± standard deviation).

	Productivity Scores	Work-Related Disability (*n* = 131)	Non-Work-Related Disability (*n* = 3)	No Disability (*n* = 31)	Disability Status *F* Value, *p* Value, df	Job Satisfaction *F* Value, *p* Value, df
**C-WPAI:GH**						
Absenteeism	1.71 ± 7.22	2.10 ± 8.04	0 ± 0	0.23 ± 1.28	*F* = 0.88; *p* = 0.42; df = 2	*F* = 15.8; ***p* < 0.0001**; df = 4
Presenteeism	26.67 ± 28.38	31.45 ± 29.14	0 ± 0	9.03 ± 15.13	*F* = 11.30; ***p* < 0.0001**; df = 2	*F* = 13.19; ***p* < 0.0001**; df = 4
Overall work impairment	27.03 ± 28.84	31.86 ± 29.69	0 ± 0	9.26 ± 15.05	*F* = 11.08; ***p* < 0.0001**; df = 2	*F* = 13.57; ***p* < 0.0001**; df = 4
Activity impairment	25.52 ± 28.53	29.85 ± 29.35	0 ± 0	9.68 ± 17.60	*F* = 9.09; ***p* = 0.0002**; df = 2	*F* = 17.33; ***p* < 0.0001**; df = 4
**C-WHO-HPQ**						
Absolute absenteeism ^a^	−0.63 ± 26.78	−0.15 ± 23.53	−1.00 ± 1.73	−2.65 ± 38.95	*F* = 1.48; *p* = 0.23; df = 2	*F* = 2.31; *p* = 0.06; df = 4
Relative absenteeism ^a^	−0.01 ± 0.12	−0.01 ± 0.10	−0.01 ± 0.01	−0.04 ± 0.17	*F* = 1.85; *p* = 0.16; df = 2	*F* = 4.03; ***p* = 0.004**; df = 4
Relative hours of work ^a^	1.01 ± 0.12	1.01 ± 0.10	1.01 ± 0.01	1.04 ± 0.17	*F* = 1.85; *p* = 0.16; df = 2	*F* = 4.03; ***p* = 0.004**; df = 4
Absolute absenteeism ^b^	−6.52 ± 31.66	−5.53 ± 27.59	0 ± 0	−11.35 ± 46.33	*F* = 2.14; *p* = 0.12; df = 2	*F* = 1.72; *p* = 0.15; df = 4
Relative absenteeism ^b^	−0.05 ± 0.17	−0.04 ± 0.16	0 ± 0	−0.09 ± 0.23	*F* = 2.29; *p* = 0.10; df = 2	*F* = 2.49; ***p* = 0.045**; df = 4
Relative hours of work ^b^	1.05 ± 0.17	1.04 ± 0.16	1 ± 0	1.09 ± 0.23	*F* = 2.29; *p* = 0.10; df = 2	*F* = 2.49; ***p* = 0.045**; df = 4
Absolute presenteeism	79.03 ± 19.85	78.40 ± 20.64	100 ± 0	79.68 ± 16.22	*F* = 1.74; *p* = 0.18; df = 2	*F* = 31.95; ***p* < 0.0001**; df = 4
Relative presenteeism	0.99 ± 0.19	0.98 ± 0.20	1 ± 0	1.06 ± 0.15	*F* = 2.50; *p* = 0.09; df = 2	*F* = 19.02; ***p* < 0.0001**; df = 4

df: degrees of freedom; ^a^ 4-week estimates; ^b^ 7-day estimates.

**Table 4:** Associations between the C-WHO-HPQ and C-WPAI:GH.

	C-WPAI:GH	Absenteeism	Presenteeism	Overall Work Impairment	Activity Impairment
C-WHO-HPQ					
Absolute absenteeism ^a^		*r*_s_ = 0.42; ***p* < 0.0001**	*r*_s_ = 0.07; *p* = 0.39	*r*_s_ = 0.08; *p* = 0.29	*r*_s_ = −0.02; *p* = 0.72
Relative absenteeism ^a^		*r*_s_ = 0.43; ***p* < 0.0001**	*r*_s_ = 0.07; *p* = 0.38	*r*_s_ = 0.08; *p* = 0.28	*r*_s_ = −0.03; *p* = 0.75
Relative hours of work ^a^		*r*_s_ = −0.43; ***p* < 0.0001**	*r*_s_ = −0.07; *p* = 0.38	*r*_s_ = −0.08; *p* = 0.28	*r*_s_ = 0.03; *p* = 0.75
Absolute absenteeism ^b^		*r*_s_ = 0.46; ***p* < 0.0001**	*r*_s_ = 0.03; *p* = 0.74	*r*_s_ = 0.04; *p* = 0.57	*r*_s_ = −0.03; *p* = 0.71
Relative absenteeism ^b^		*r*_s_ = 0.47; ***p* < 0.0001**	*r*_s_ = 0.03; p = 0.73	*r*_s_ = 0.05; *p* = 0.57	*r*_s_ = −0.03; *p* = 0.72
Relative hours of work ^b^		*r*_s_ = −0.47; ***p* < 0.0001**	*r*_s_ = −0.03; *p* = 0.73	*r*_s_ = −0.05; *p* = 0.57	*r*_s_ = 0.03; *p* = 0.72
Absolute presenteeism		*r*_s_ = −0.32; ***p* < 0.0001**	*r*_s_ = −0.48; ***p* < 0.0001**	*r*_s_ = −0.48; ***p* < 0.0001**	*r*_s_ = −0.45; ***p* < 0.0001**
Relative presenteeism		*r*_s_ = −0.29; ***p* = 0.0002**	*r*_s_ = −0.11; *p* = 0.16	*r*_s_ = −0.11; *p* = 0.17	*r*_s_ = −0.11; *p* = 0.17

^a^ 4-week estimate; ^b^ 7-day estimates.

**Table 5:** Factors correlated with the C-WPAI:GH using Bayesian model averaging (regression coefficient β and 95% credible interval (95% CrI)).

	C-WPAI:GH			
	Absenteeism	Presenteeism	Overall Work Impairment	Activity Impairment
Age		0.52 (0.09; 0.95)	0.53 (0.10; 0.96)	0.45 (0.02; 0.88)
Gender (female vs. male)				
Education (vs. junior high school)				
Senior high school	8.81 (0.22; 17.40)			29.94 (6.46; 53.41)
Undergraduate	9.13 (0.47; 17.79)			29.28 (5.62; 52.95)
Postgraduate	8.21 (−1.03; 17.44)			20.81 (−4.42; 46.05)
Job (vs. manager, professional)				
Disability status (vs. work-related disability) Non-work-related disability No disability				
Job satisfaction				
(vs. completely dissatisfied)				
Moderately dissatisfied	−9.06 (−14.04; −4.09)			
Neither satisfied nor dissatisfied	−10.58 (−15.90; −5.25)			
Moderately satisfied	−10.27 (−15.65; −4.90)			
Completely satisfied	−9.41 (−16.06; −2.76)			
Physical functioning	−0.41 (−0.59; −0.22)			
Role—physical				
Role—emotional				
Bodily pain		−0.56 (−0.75;−0.36)	−0.56 (−0.76; −0.36)	−0.45 (−0.65; −0.25)
Social functioning		−0.33 (−0.57; −0.09)	−0.33 (−0.57; −0.08)	−0.50 (−0.74; −0.25)
Mental health				
Vitality				
General health		−0.27 (−0.50; −0.04)	−0.27 (−0.50; −0.04)	−0.30 (−0.53; −0.07)

## Data Availability

Not applicable.

## Supplementary Materials

The following supporting information can be downloaded at: https://www.mdpi.com/article/10.3390/ijerph19074417/s1. The Traditional Chinese Version of Work Productivity and Activity Impairment Questionnaire: General Health (C-WPAI:GH) [11]. The Traditional Chinese Version of the World Health Organization’s Health and Work Performance Questionnaire (C-WHO-HPQ) [1,[2]]. Table S1: Comparison of mean C-WPAI:GH scores among disability status groups. Table S2: Comparison of mean C-WPAI:GH scores among job satisfaction groups. Table S3: Comparison of mean C-WHO-HPQ scores among job satisfaction groups.
