# Supplementary material for: A Comparison of the Validities of Traditional Chinese Versions of the Work Productivity and Activity Impairment Questionnaire: General Health and the World Health Organization’s Health and Work Performance Questionnaire
Source: Int J Environ Res Public Health. 2022 Apr 6;19(7):4417. doi: 10.3390/ijerph19074417 (PMC8998541; doi:10.3390/ijerph19074417)
Supplement: Supplementary file 1 [file ijerph-19-04417-s001.zip › ijerph-1644361-supplementary.pdf]

Supplemental Materials

The Traditional Chinese Version of Work Productivity and Activity Impairment Questionnaire: General Health (C-WPAI:GH) 2
The Traditional Chinese Version of the World Health Organization's Health and Work Performance Questionnaire (C-WHO-HPQ) 3
Table S1. Comparison of Mean C-WPAI:GH Scores among Disability Status Groups. 5
Table S2. Comparison of Mean C-WPAI:GH Scores among Job satisfaction Groups. 5
Table S3. Comparison of Mean C-WHO-HPQ Scores among Job satisfaction Groups. 6

The Traditional Chinese Version of Work Productivity and Activity Impairment Questionnaire: General Health (C-WPAI:GH) [11]

下列問題是要詢問您的健康問題對您的工作和日常活動能力的影響。所謂健康問題是指任何生理或情緒的問題或症狀。*請根據指示填寫答案或圈選一個數字。*

1) 您目前有工作嗎（有薪工作）？ ____ 沒有 ____ 有

如果答案為沒有，請勾選「沒有」並跳到問題 6。

下面的問題是關於**過去七天**中的情況，不包括今天。

2) 在過去七天中，您由於健康問題而缺勤多少小時？*請包括您由於健康問題請病假、上班遲到或提前下班而缺勤的時數，但是不包括由於參加本研究而缺勤的時間。*

__________小時

3) 在過去七天中，您有任何其他原因，例如度假、節假日、請假參加本研究而缺勤多少小時？

__________小時

4) 在過去七天中，您實際工作多少小時？

__________小時*（如果是「0」，請跳到問題 6。）*

5) 在過去七天中，您的健康問題對於您在工作時的工作效率有多大影響？

請考慮您在能夠完成的工作量或能夠做的工作種類方面受到限制的日子、您完成的工作量少於自己的期望的日子，或是您不能像往常那樣細心工作的日子。如果健康問題對您的工作影響很小，選擇一個較小的數字。如果健康問題對您的工作影響很大，選擇一個較大的數字。

請僅考慮健康問題對您在工作時的生產力的影響程度。

健康問題對我的工作沒有影響	0	1	2	3	4	5	6	7	8	9	10	健康問題使我完全 不能工作

圈選一個數字

6) 在過去七天中，您的健康問題對於您工作以外進行日常活動的能力有多大影響？

日常活動意指您通常進行的活動，如做家務、購物、照看小孩、體育鍛鍊、學習等。請考慮您在活動量或能夠做的活動種類方面受到限制的時候，以及您完成的活動量少於自己的期望的時候。如果健康問題對您的活動影響很小，選擇一個較小的數字。如果健康問題對您的活動影響很大，選擇一個較大的數字。

請僅考慮健康問題對於您工作以外的日常活動能力的影響程度。

健康問題對我的日常活動沒有影響	0	1	2	3	4	5	6	7	8	9	10	健康問題使我完全不能進行日常活動

圈選一個數字

The Traditional Chinese Version of the World Health Organization's Health and Work Performance Questionnaire (C-WHO-HPQ) [1,2]

B3. 過去 7 天，你總共工作了多少小時?

(如果超過 97 小時，請輸入 97 小時。)

□□

小時數(00-97)

B4. 您的雇主一般希望您每週 7 天工作多少小時?

(如果相關預期並不固定，請估計平均數。 如果超過 97 小時，請輸入 97 小時。)

□□

小時數(00-97)

B5.現在，請回想一下您在過去 4 周（28 天）的工作經歷。在下節空白處，寫出你在以下每一種工作狀態下度過的天數。

**在過去 4 周（28 天）中，有多少天數符合以下描述?**	**天數 (00-28)**
B5a. ...因為您本身的身體或精神健康問題而缺勤了**一整個工作天?** (請只考慮因**您自身**健康而缺勤的日數，而非別人的健康。)	□□
B5b. ...因為任何其他原因(包括特修)而缺勤了**一整個工作天?**	□□
B5c. ...因為身體或精神健康問題而缺勤**幾個小時（不到一天）?** (請只考慮因**您自身**健康而缺勤的日數，而非別人的健康。)	□□
B5d. ...因為任何其他原因(包括特修)而缺勤**幾個小時（不到一天）?**	□□
B5e. ...早到、晚歸或在休假日工作**?**	□□

B6.在過去的4周（28天）裡，您總共工作了多少小時？（請看以下例子）

□□□ 過去4周（28天）的工作小時數。

過去4周工作小時數計算範例

每周40小時，為期4周=160小時

每周35小時，為期4周=140小時

每周40小時，為期4周；缺勤2天(每天8小時計)=144小時。

每週40小時，為期4周；部分缺勤3天(每天4小時計)=148小時。

每週35小時，為期4周；缺勤2天(每天8小時計)，加上部分缺勤3天(每天4小時計)=112小時。

B9. 在0到10的範圍內，0是在您的職位中任何人可能呈現之最差工作表現，10則是最佳員工的表現，你如何評價與你類似職位的其他大多數員工的平時表現？

最差 **表現**										***最佳*** **表現**
0	1	2	3	4	5	6	7	8	9	10
○	○	○	○	○	○	○	○	○	○	○

B10. 使用同樣的0到10分的評分標準，您會如何評價您在過去一兩年中的平時工作表現？

最差 **表現**										***最佳*** **表現**
0	1	2	3	4	5	6	7	8	9	10
○	○	○	○	○	○	○	○	○	○	○

B11. 使用同樣的0到10分的評分標準，您如何評價您在過去4周（28天）的整體工作表現？

最差 **表現**										***最佳*** **表現**
0	1	2	3	4	5	6	7	8	9	10
○	○	○	○	○	○	○	○	○	○	○

Table S1. Comparison of Mean C-WPAI:GH Scores among Disability Status Groups.

Groups Compared	Difference in Means	Adjusted P
Presenteeism		
Group 2 – 1	-31.591	0.1100
Group 3 – 1	-23.924	**0.0001**
Group 3 - 2	7.667	0.8839
Overall work impairment		
Group 2 – 1	-31.994	0.1122
Group 3 – 1	-24.090	**0.0001**
Group 3 - 2	7.905	0.8810
Activity impairment		
Group 2 – 1	-30.000	0.1455
Group 3 – 1	-21.667	**0.0004**
Group 3 - 2	8.333	0.8687

Group 1: Work-related disability; Groupt 2: Non-work-related disability; Group 3: No disability

Table S2. Comparison of Mean C-WPAI:GH Scores among Job satisfaction Groups.

Groups Compared	Difference in Means	Adjusted P
Absenteeism		
Group 2 – 1	-14.081	**< 0.0001**
Group 3 – 1	-18.395	**< 0.0001**
Group 4 – 1	-17.917	**< 0.0001**
Group 5 – 1	-18.469	**< 0.0001**
Group 3 – 2	-4.314	**0.0336**
Group 4 – 2	-3.836	0.0982
Group 5 – 2	-4.388	0.3369
Group 4 – 3	0.478	0.9930
Group 5 – 3	-0.074	0.9999
Group 5 – 4	-0.552	0.9999
Presenteeism		
Group 2 – 1	-25.901	0.1183
Group 3 – 1	-54.125	**< 0.0001**
Group 4 – 1	-46.746	**< 0.0001**
Group 5 – 1	-65.857	**< 0.0001**
Group 3 – 2	-28.224	**< 0.0001**
Group 4 – 2	-20.845	**0.0085**
Group 5 – 2	-39.957	**0.0004**
Group 4 – 3	7.379	0.4740
Group 5 – 3	-11.732	0.6323
Group 5 – 4	-19.111	0.1747
Overall work impairment		
Group 2 – 1	-25.690	0.1322
Group 3 – 1	-55.119	**< 0.0001**
Group 4 – 1	-47.492	**< 0.0001**
Group 5 – 1	-66.898	**< 0.0001**
Group 3 – 2	-29.429	**< 0.0001**
Group 4 – 2	-21.802	**0.0059**
Group 5 – 2	-41.208	**0.0003**
Group 4 – 3	7.627	0.4528

Groups Compared	Difference in Means	Adjusted P
Group 5 – 3	-11.779	0.6399
Group 5 – 4	-19.406	0.1725
Activity impairment		
Group 2 – 1	-21.988	0.2204
Group 3 – 1	-56.519	**< 0.0001**
Group 4 – 1	-48.598	**< 0.0001**
Group 5 – 1	-66.857	**< 0.0001**
Group 3 – 2	-34.532	**< 0.0001**
Group 4 – 2	-26.610	**0.0002**
Group 5 – 2	-44.870	**< 0.0001**
Group 4 – 3	7.921	0.3667
Group 5 – 3	-10.338	0.7108
Group 5 – 4	-18.259	0.1856

Group 1: Completely dissatisfied; Group 2: Moderately dissatisfied; Group 3: Neither satisfied nor dissatisfied; Group 4: Moderately satisfied; Group 5: Completely satisfied

Table S3. Comparison of Mean C-WHO-HPQ Scores among Job satisfaction Groups.

Groups Compared	Difference in Means	Adjusted P
Relative absenteeism [a]		
Group 2 – 1	-0.101	0.2319
Group 3 – 1	-0.140	**0.0166**
Group 4 – 1	-0.162	**0.0040**
Group 5 – 1	-0.106	0.3187
Group 3 – 2	-0.039	0.5990
Group 4 – 2	-0.061	0.1943
Group 5 – 2	-0.004	0.9999
Group 4 – 3	-0.022	0.8172
Group 5 – 3	0.035	0.8924
Group 5 – 4	0.057	0.5902
Relative hours of work [a]		
Group 2 – 1	0.101	0.2319
Group 3 – 1	0.140	**0.0166**
Group 4 – 1	0.162	**0.0040**
Group 5 – 1	0.106	0.3187
Group 3 – 2	0.039	0.5990
Group 4 – 2	0.061	0.1943
Group 5 – 2	0.004	0.9999
Group 4 – 3	0.022	0.8172
Group 5 – 3	-0.035	0.8924
Group 5 – 4	-0.057	0.5902
Relative absenteeism [b]		
Group 2 – 1	-0.170	0.1381
Group 3 – 1	-0.181	0.0560
Group 4 – 1	-0.210	**0.0187**
Group 5 – 1	-0.160	0.3101
Group 3 – 2	-0.011	0.9987
Group 4 – 2	-0.040	0.8733
Group 5 – 2	0.011	0.9998
Group 4 – 3	-0.030	0.8746
Group 5 – 3	0.022	0.9955

Groups Compared	Difference in Means	Adjusted P
Group 5 – 4	0.051	0.9057
Relative hours of work [b]		
Group 2 – 1	0.170	0.1381
Group 3 – 1	0.181	0.0560
Group 4 – 1	0.210	**0.0187**
Group 5 – 1	0.160	0.3101
Group 3 – 2	0.011	0.9987
Group 4 – 2	0.040	0.8733
Group 5 – 2	-0.011	0.9998
Group 4 – 3	0.030	0.8746
Group 5 – 3	-0.022	0.9955
Group 5 – 4	-0.051	0.9057
Absolute presenteeism		
Group 2 – 1	33.478	**< 0.0001**
Group 3 – 1	50.845	**< 0.0001**
Group 4 – 1	56.296	**< 0.0001**
Group 5 – 1	67.000	**< 0.0001**
Group 3 – 2	17.367	**< 0.0001**
Group 4 – 2	22.818	**< 0.0001**
Group 5 – 2	33.522	**< 0.0001**
Group 4 – 3	5.451	0.2639
Group 5 – 3	16.155	**0.0145**
Group 5 – 4	10.704	0.2363
Relative presenteeism		
Group 2 – 1	0.331	**< 0.0001**
Group 3 – 1	0.460	**< 0.0001**
Group 4 – 1	0.515	**< 0.0001**
Group 5 – 1	0.504	**< 0.0001**
Group 3 – 2	0.129	**0.0101**
Group 4 – 2	0.184	**< 0.0001**
Group 5 – 2	0.172	**0.0441**
Group 4 – 3	0.055	0.3281
Group 5 – 3	0.044	0.9312
Group 5 – 4	-0.012	0.9996

[a] 4-week estimates; [b] 7-day estimates; Group 1: Completely dissatisfied; Group 2: Moderately dissatisfied; Group 3: Neither satisfied nor dissatisfied; Group 4: Moder-ately satisfied; Group 5: Completely satisfied
